# Effects of Metformin Treatment on Homocysteine Levels and Metabolic Parameters of Women With Polycystic Ovary Syndrome 

**Published:** 2015-11

**Authors:** Soheila Riahinejad, Ahmad Mirdamadi, Elham Alizadeh

**Affiliations:** Medical School, Islamic Azad University, Najafabad Branch, Isfahan, Iran

**Keywords:** Metformin, Homocysteine, Polycystic Ovary Syndrome, Metabolic Parameters

## Abstract

**Objective:** Polycystic ovary syndrome (PCOS) is one of the most common endocrine disorders in women. Metformin is a biguanide commonly used to improve PCOS symptoms. Effect of metformin on the levels of serum homocysteine (Hcy) in PCOS women is unclear. The aim of this study is evaluating the effect of metformin administration on serum Hcy levels and metabolic parameters of PCOS patients.

**Materials and methods:** Thirty three patients with PCOS were enrolled in this study who were selected randomly. All patients received metformin from the fifth day of menstrual cycle at a dose of 850 mg (one tablet daily) for 3 months. Body mass index, Triglycerides, total cholesterol, high-density lipoprotein cholesterol (HDL-C), low-density lipoprotein cholesterol (LDL-C), fasting blood sugar and homocysteine levels were recorded at entry into the study and after 3 months treatment.

**Results:** BMI, plasma Homocysteine concentrations and fasting blood sugar levels were significantly (p < 0.05) decreased after the treatment period. No significant changes were observed in the lipid profiles of patients. There was a weak negative correlation between homocysteine and LDL cholesterol serum levels (p = 0.04, r = -0.27).

**Conclusion:** Treatment with metformin in PCOS women may lead to beneficial effects in terms of BMI, plasma homocysteine concentrations and fasting blood sugar with no remarkable effect on lipid profile.

## Introduction

Polycystic ovary syndrome (PCOS) is one of the most common endocrine disorders in female characterized by chronic menstrual dysfunction, clinical or biochemical signs of hyperandrogenism, and polycystic ovaries detected by ultrasound (1). The prevalence of PCOS is as high as 6%–9% base on the National Institutes of Health (NIH) criteria (2). More than half of women with PCOS are affected by a number of co morbidities including metabolic syndrome, hypertension, dyslipidemia, impaired glucose tolerance, and type 2 diabetes mellitus due to Insulin resistance (2). 

Compensatory hyperinsulinemia in these patients is related to elevated homocysteine levels via suppression of cystathione beta synthase. Excess Hcy in the blood stream may play a role in the cardiovascular excess risk associated with PCOS due to arterial vessels injures (3). Elevated Hcy is also related to pregnancy complications and neonatal defects such as pre-eclampsia and Congenital heart defects (4-6). Bioavailability of folic acid, B vitamins, and methyl group donors is also affects homocysteine (Hcy) metabolism (7). 

Metformin is a biguanide commonly used to improving insulin sensitivity and have insulin-lowering effects. Therefore, this agent have a role in the treatment of PCOS by reducing insulin resistance (8, 9). Recent data suggest that metformin improves ovulation rate, clinical pregnancy rate and live-birth rates in women with polycystic ovary syndrome (10-12). However, in certain clinical situations, in patients with type 2 diabetes, metformin increase serum homocysteine (Hcy) levels may be by inducing malabsorption of vitamin B12 and Folic acid and reducing their serum levels (13, 14).

Dissimilar the response of serum Hcy levels to the consumption of metformin in diabetic patients, effect of metformin on the levels of serum Hcy in PCOS women is unclear, may be due to heterogeneity of the studies (15-17).

The aim of this study is evaluating the effect of metformin administration on serum Hcy levels and metabolic parameters of patients with PCOS.

## Materials and methods

This study was a prospective before - after clinical trial, without control group, designed to evaluate the effects of 3 months treatment with metformin on homocysteine Levels and metabolic parameters of women with Polycystic Ovary Syndrome . This trial was conducted in various clinics in Isfahan (Iran’s third largest city, located in the center of Iran), Iran. The Medical Ethics Committee of Isfahan University of Medical Sciences has approved the study design, protocols and informed consent procedure (Reference code: 493053).

Thirty three patients with PCOS were enrolled in this study that was selected randomly .The diagnosis of PCOS was based on the National Institutes of Health (NIH) criteria (8). The following general exclusion criteria were considered: age < 20 or > 35 years, renal disorders, diabetes, current or previous (within the last 3 months) use of metformin. The sample size was calculated on the assumption that a difference of 0.22(µmol/l) in homocysteine level is relevant, with α = 0.05 and power = 80%. We considered 10% attrition rate and the final sample size was estimated 33 patients. 

At entry into the study all participants underwent clinical assessments that consisted of anthropometric measurements, which included height, weight, BMI (ratio between the weight and the square of the height). Also, for baseline biochemical assessment, During the second to fifth day of menstrual bleeding, 15 milliliters venous blood sample were obtained from each patients after fasting for 12 -14 hours and resting in bed, to assess lipid profile (Triglycerides, total cholesterol, high-density lipoprotein cholesterol (HDL-C), low-density lipoprotein cholesterol (LDL-C)), fasting blood sugar and homocysteine level .

All participants received metformin (metformin tablet, 500 mg, Aria©, Iran) from the fifth day of menstrual bleeding at a dose of 500 mg (one tablet daily before lunch) for 3 month. 

After 3 months of treatment, all participants underwent clinical and biochemical evaluations in the same way of first assessments at entry into the study.

All statistical analysis was performed by using SPSS version 20 for windows. Findings have shown as relative frequencies, mean and standard deviation. The differences in quantitative variables before and after treatment were assessed by paired T test. Pearson correlation used to determine the relationship between variables. P values less than 0.05 is considered significant.

## Results

A total of 33 patients were enrolled in this study, all of whom were included in the clinical and biochemical evaluations and received metformin (850 mg/daily). Thirty patients (90%) completed 3 months of treatment. The mean age of patient was 25.5 years (standard deviation = 3.48) and their mean weight was 63.70 kg (standard deviation = 8.73). Table 1 summarizes the clinical and biochemical characteristics of patients at baseline and 3 months of treatment. Significant (p < 0.05) decrease was observed in the level of homocysteine, Fasting blood sugar, BMI and weight. At the end of the study, no significant changes (p > 0.05) had been observed in the lipid profile of patients (Table 1). Comparison of homocysteine levels in women with BMI < 25 and women with BMI >= 25 treated with metformin at study entry and after 3 months of treatment was shown in table 2.

In the Pearson correlation test, no correlation was found between Hcy levels and BMI, total cholesterol, HDL cholesterol, triglycerides, fasting blood sugar (p > 0.05). There was a correlation between homocysteine levels and LDL cholesterol (p = 0.04, r = -0.27) before and after treatment (table 3). This correlation was negative (increasing one decreases the other) and weak because r was small (under 0.4).

**Table 1:** Clinical and biochemical characteristics in PCOS patients treated with metformin at study entry and after 3 months of treatment

	Baseline Mean (SD)	After treatment Mean (SD)	p value
**BMI (kg/m2)**	**23.97 (3.25)**	**23.71 (3.37)**	**0.035**
**Homocysteine (µmol/l)**	**9.11 (2.7)**	**8.55 (2.49)**	**0.008**
**Total cholesterol (mg/dl)**	**112.57 (17.97)**	**112.07 (17.64)**	**0.187**
**HDL cholesterol (mg/dl)**	**32.70 (6.00)**	**33.10 (6.25)**	**0.262**
**LDL cholesterol (mg/dl)**	**82.87 (7.66)**	**81.93 (7.64)**	**0.152**
**Triglycerides (mg/dl)**	**98.87 (19.94)**	**98.47 (19.93)**	**0.552**
**Fasting blood sugar (mg/dl)**	**81.03 (5.8)**	**78.43 (5.30)**	**0.011**

**Table 2:** Comparison of homocysteine levels in women with BMI<25 and women with BMI > =25 treated with metformin at study entry and after 3 months of treatment

	BMI < 25 n = 21			BMI >= 25 n = 9		
	Baseline Mean (SD)	After treatment Mean (SD)	p value	Baseline Mean ( SD)	After treatment Mean ( SD)	p value
**Homocysteine (µmol/l)**	**9.15 (2.34)**	**8.18 (2.24)**	**0.02**	**9.78 (3.48)**	**9.39 (2.97)**	**0.22**

**Table 3:** The Pearson correlations between Homocysteine level and other parameters before and after treatment

	**Baseline** **Homocysteine** **(Pearson Correlation)**	**p value**	**3 months** **characteristics**	**3 months** **Homocysteine** **(Pearson Correlation)**	**p value**
BMI	0.032	0.432	BMI	0.163	0.194
Total cholesterol	0.053	0.391	Total cholesterol	0.004	0.49
HDL cholesterol	0.06	0.36	HDL cholesterol	0.154	0.20
LDL cholesterol	-0.276	0.040	LDL cholesterol	-0.276	0.04
Triglycerides	-0.132	0.243	Triglycerides	-0.176	0.176
Fasting blood sugar	0.062	0.373	Fasting blood sugar	-0.237	0.10

## Discussion

This study provides an experimental evidence that 3 months of metformin therapy result in a significant decrease in BMI, plasma homocysteine concentrations and Fasting blood sugar without significant changes in lipid profile parameters.

Some studies on the effect of metformin on BMI in PCO women are similar to our findings (18, 19). However, other studies failed to demonstrate any significant BMI reduction during the treatment period (15, 17, 20).

In agreement with previous data, in our study Fasting blood sugar level were significantly differ from base line. Studies shows metformin deceases hepatic glucose output and corrects the response to oral glucose tolerance (15-17).

One of the important findings of our study is that there is a significant decrease in serum Hcy levels after 3 months administration of metformin. In agreement with our finding, Schachter et al reports decreases in Hcy levels after treatment with metformin for periods of 6–16 weeks (16). Our findings are not confirm by some studies who reports that there are no change or increase in the concentration of plasma Hcy after metformin treatment in PCOS (17,18), also treatment of patients with Diabetes Mellitus II (DM II) with metformin increased the level of Hcy (21).The discrepancies in these studies may be due to differences in the patient characteristics, especially their BMI. Most of studies that shows no changes or decrease in plasma Hcy, conducted in lean PCOS women .The mean BMI of our patients is 23.97(SD = 3.25) (kg/m2) and Comparison of homocysteine level change in women with BMI < 25 and women with BMI >= 25 after metformin treatment demonstrate the significant decrease only in women with BMI < 25 and had no effect on over weight and obese women’s Hcy level, although, it must be interpreted cautiously, because of the small sample size. However, it may be conclude that the metformin consumption decreases the level of Hcy in lean PCOS women and in other words, the effect of metformin on plasma Hcy level is base on patient BMI. 

We have found that 3 months of metformin treatment not significantly changed serum lipids and lipoprotein levels in women with PCOS, in accordance with some previous studies (22). An another studies have shown reduction in triglyceride levels and increase in HDL by metformin treatment in women with PCOS compared with controls (23, 24). A small number of patients, differences in the patient characteristics and differences in the treatment periods may explain these discrepancies.

In our study, we suggest a weak negative correlation between Hcy level and LDL before and after the treatment similar to other studies (3, 19).

Finally, further studies with larger sample size are needed to clarify the effect of metformin consumption in lean and obese PCOS women separately.

In conclusion, 3 months of metformin therapy results in beneficial effects in PCOS women in terms of BMI, plasma homocysteine concentrations and Fasting blood sugar, without any remarkable effect on lipid profile.
